# Synthesis of ZnMn_2_O_4_ Nanoparticles by a Microwave-Assisted Colloidal Method and their Evaluation as a Gas Sensor of Propane and Carbon Monoxide

**DOI:** 10.3390/s18030701

**Published:** 2018-02-27

**Authors:** Juan Pablo Morán-Lázaro, Erwin Said Guillen-López, Florentino López-Urias, Emilio Muñoz-Sandoval, Oscar Blanco-Alonso, Héctor Guillén-Bonilla, Alex Guillén-Bonilla, Verónica María Rodríguez-Betancourtt, Marciano Sanchez-Tizapa, María de la Luz Olvera-Amador

**Affiliations:** 1 Department of Computer Science and Engineering, CUValles, University of Guadalajara, Ameca, Jalisco 46600, Mexico; alex.guillen@profesores.valles.udg.mx; 2 Department of Natural and Exact Sciences, CUValles, University of Guadalajara, Ameca, Jalisco 46600, Mexico; erwin.guillen@alumnos.udg.mx (E.S.G.-L.); msanchez@profesores.valles.udg.mx (M.S.-T.); 3 Advanced Materials Department, IPICYT, San Luis Potosí 78216, Mexico; flo@ipicyt.edu.mx (F.L.-U.); ems@ipicyt.edu.mx (E.M.-S.); 4 Department of Physics, CUCEI, University of Guadalajara, Guadalajara, Jalisco 44410, Mexico; oscar.blanco@cucei.udg.mx; 5 Department of Project Engineering, CUCEI, University of Guadalajara, Guadalajara, Jalisco 44410, Mexico; hguillenbonilla@gmail.com; 6 Department of Chemistry, CUCEI, University of Guadalajara, Guadalajara, Jalisco 44410, Mexico; veronica.rodriguez@red.cucei.udg.mx; 7 Department of Electrical Engineering (SEES), CINVESTAV-IPN, Mexico City 07360, Mexico; molvera@cinvestav.mx

**Keywords:** ZnMn_2_O_4_, nanoparticles, microwave, gas sensor

## Abstract

Spinel-type ZnMn_2_O_4_ nanoparticles were synthesized via a simple and inexpensive microwave-assisted colloidal route. Structural studies by X-ray diffraction showed that a spinel crystal phase of ZnMn_2_O_4_ was obtained at a calcination temperature of 500 °C, which was confirmed by Raman and UV-vis characterizations. Spinel-type ZnMn_2_O_4_ nanoparticles with a size of 41 nm were identified by transmission electron microscopy. Pellet-type sensors were fabricated using ZnMn_2_O_4_ nanoparticles as sensing material. Sensing measurements were performed by exposing the sensor to different concentrations of propane or carbon monoxide at temperatures in the range from 100 to 300 °C. Measurements performed at an operating temperature of 300 °C revealed a good response to 500 ppm of propane and 300 ppm of carbon monoxide. Hence, ZnMn_2_O_4_ nanoparticles possess a promising potential in the gas sensors field.

## 1. Introduction

The excessive emission of polluting and toxic gases, such as propane (C_3_H_8_) and carbon monoxide (CO), in urban and rural areas because of human activities such as transportation, production, landfill, and livestock farming has generated many public health concerns as well as environmental concerns over global warming. In this sense, resistive sensors based on semiconductor metal oxides have been widely used for the detection of several gases and vapors. These types of gas sensors have been used because they are cheap, easy to operate, and chemically stable in harsh conditions. The operational principle of these sensors is based on changes of the electrical conductivity when a reaction occurs between pre-adsorbed oxygen molecules, or the material itself, and a reducing or oxidizing gas [1]. Most of these sensors are based on binary metal oxides, such as SnO_2_ [2,3,4], ZnO [5,6], and TiO_2_ [7,8]. More recently, ternary compounds such as NiSb_2_O_6_ [9], CuSb_2_O_6_ [10], MgSb_2_O_6_ [11], LaCoO_3_ [12], ZnM_2_O_4_ (M = Fe, Co, Cr) [13], and MFe_2_O_4_ (M = Co, Ni) [14], have also been used. Ternary metallic oxides with a particle size on the nanometric scale display significant responses when they are exposed to reducing or oxidizing gases. As is already known, when the particle size is reduced to a nanometric size, the surface area-to-volume ratio increases, favoring the adsorption of a gas on the sensor [15]. Also, it has been reported that the gas response depends on the morphology of the material, the gas concentration, and the operational temperature [12,16,17,18]. In fact, some common issues of resistive sensors based on metallic oxides are the low response and the high operating temperature; therefore, the search for new materials without these problems is an important research topic.

Among the spinel-type oxides, the zinc manganite (ZnMn_2_O_4_) is a compound that possesses a normal spinel structure, where the divalent Zn cation occupies the tetrahedral site and the trivalent Mn cation occupies the octahedral site in the cubic spinel structure. ZnMn_2_O_4_ is an interesting compound that has been widely used as an electrode for Li-ion batteries due to its low cost and being environmentally friendly [19,20,21,22,23,24]. Also, this material has been used as a supercapacitor [25,26,27,28,29] and thermistor [30] due to its structural, physical, and chemical properties. It has been reported that zinc manganite shows strong catalytic activity [31,32,33,34,35]; therefore, this material could perform well as a gas sensor. To date, ZnMn_2_O_4_ has been little studied in the gas sensors field. Nassar et al. [36] developed a chemical sensor based on ZnMn_2_O_4_ nanostructures, which exhibited high sensitivity to omeprazole and lansoprazole drugs. Sorita and Kawano [37] used the oxide as ann electrode in a CO sensor based on a zirconia galvanic cell, obtaining a response of 9.68 at 2000 ppm of CO at 400 °C. Na et al. [38], as well as Panmatarith and Innoi [39], studied the response of sensors made from ZnO–ZnMn_2_O_4_ nanostructures to substances like ethanol, ammonia, carbon monoxide, propane, hydrogen peroxide, etc. 

In regard to the synthesis of nanometric ZnMn_2_O_4_, several routes have been commonly employed: polymer-pyrolysis [23], solid-state reaction [24], sol-gel [31], co-precipitation [33], hydrothermal [29,40], and solvothermal methods [41]. Reports on the synthesis of ZnMn_2_O_4_ nanoparticles using a microwave-assisted route are still limited. Recently, Brahma et al. [42] obtained ZnMn_2_O_4_ nanoparticles using a three-step process that included microwave irradiation, centrifugation, and washing. 

In this work, nanoparticles of the ternary compound ZnMn_2_O_4_ were synthesized using a microwave-assisted colloidal route without the use of a centrifugation and washing [9,10,43,44]. It is noteworthy to mention that microwave irradiation provides a rapid evaporation of the solvent from the precursor solutions, shortening the reaction times [45]. The ZnMn_2_O_4_ nanoparticles were characterized by means of thermogravimetric analysis (TGA), X-ray powder diffraction (XRD), Raman and UV-vis spectroscopies, and transmission electron microscopy (TEM). In addition, the performance of zinc manganite nanoparticles as a sensor of C_3_H_8_ and CO was evaluated. 

## 2. Materials and Methods

### 2.1. ZnMn_2_O_4_ Synthesis

**Figure 1:**
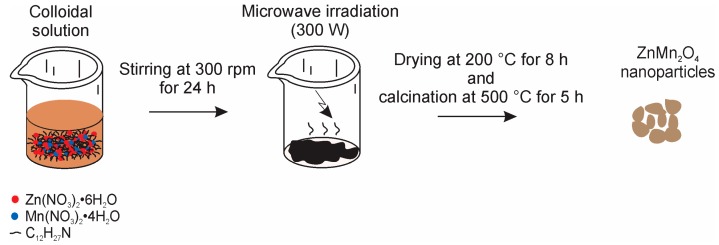
Schematic representation of the synthesis of ZnMn_2_O_4_ nanoparticles.

ZnMn_2_O_4_ nanoparticles were prepared by a microwave-assisted colloidal method, using as starting reagents: Zinc nitrate hexahydrate (Zn(NO_3_)_2_·6H_2_O, Sigma-Aldrich 98%, St. Louis, MO, USA), manganese(II) nitrate tetrahydrate (Mn(NO_3_)_2_·4H_2_O, Aldrich 99%, St. Louis, MO, USA), dodecylamine (C_12_H_27_N, Aldrich 98%, St. Louis, MO, USA), and ethanol (C_2_H_6_O, Hycel 96%, Zapopan, Jalisco, Mexico). Three solutions were prepared using 5 mL of ethanol as solvent: (i) A 5 mmol solution of zinc nitrate; (ii) a 10 mmol solution of manganese nitrate; (iii) a 16.2 mmol solution of dodecylamine. All the solutions were stirred at ~300 rpm for 20 min at room temperature. Afterwards, the zinc nitrate solution was slowly added under stirring to the dodecylamine solution. Subsequently, the manganese solution was added dropwise to the zinc + dodecylamine mixture, producing a brownish solution with pH = 2. To prevent gelation of the solution, 8 mL of ethanol were added. The final solution was kept under stirring at ~300 rpm for 24 h at room temperature. After that, microwave radiation was applied for periods of 2 min for about 4 h until the solvent was removed. A black viscous material was obtained. For our purposes, a kitchen-type microwave oven (Whirlpool WM1311S, Benton Harbor, MI, USA) was used, operating at low power (~300 W). The resulting material was dried in air at 200 °C for 8 h using a muffle (Terlab TE-M20DR, Zapopan, Jalisco, Mexico). The calcination of the resulting black powder was carried out at different temperatures in the range from 300 to 600 °C for 5 h, at a heating rate of 100 °C/h. The synthesis process is depicted in Figure 1. 

### 2.2. Characterization of ZnMn_2_O_4_ Powders

The thermal decomposition of the precursor powder, as a function of temperature, was followed at a rate of 10 °C/min by means of a thermogravimetric analysis using a PerkinElmer TGA 4000 device in an atmosphere of air. The crystal structure of the calcined samples was analyzed by the XRD technique using a PANalytical Empyrean equipment operated at 45 kV and 40 mA, with CuKα and λ = 1.546 Å. The XRD data were collected at room temperature in a 2θ range from 10° to 70° with steps of 0.02°. The main Raman vibrational modes of the zinc manganite were obtained using a Thermo Scientific DXR confocal Raman microscope (λ = 633 nm). The Raman spectrum was recorded from 100 to 800 cm^−1^ at room temperature, using an exposure time of 60 s and a laser power of 5 mW. The absorbance was measured with a Shimadzu UV3600 spectrophotometer in the range 200–800 nm. The ZnMn_2_O_4_ nanoparticles were analyzed by TEM and high-resolution transmission electron microscopy (HRTEM) using a FEI Tecnai-F30 system operated at an acceleration voltage of 300 kV. 

### 2.3. Gas Sensitivity Tests

**Figure 2:**
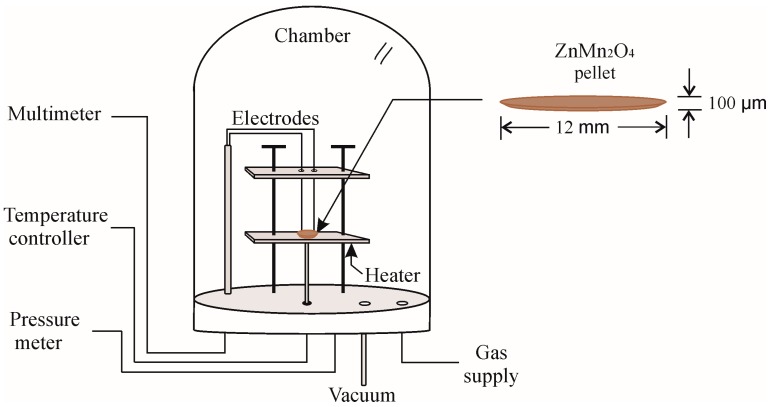
Schematic of the system used for gas sensitivity measurements.

Pellet-type sensors based on ZnMn_2_O_4_ nanoparticles were used for the detection of C_3_H_8_ and CO. For the fabrication of the pellets, with a thickness of ~100 µm and a diameter of 12 mm, the ZnMn_2_O_4_ powder calcined at 500 °C was compacted with a uniaxial force of 10 tons for 5 min using a manual pressure machine (Simplex Ital Equip). The pellet was placed in a vacuum chamber at a vacuum level of 10^−3^ torr, partial pressure and gas concentration within the chamber were controlled using a TM20 Leybold detector. The electrical resistance of the ZnMn_2_O_4_ sensor exposed to the gases was measured using a digital multimeter (Keithley 2001, Cleveland, OH, USA). A gas sensing system such as the one shown in Figure 2 was used. The sensor response has been usually defined as *β* = *R*_a_/*R*_g_ for reducing gases, where *R*_a_ is the resistance in the air and *R*_g_ is the resistance in the sampled gas [46,47,48]. 

## 3. Results and Discussion

### 3.1. Thermogravimetric Analysis

**Figure 3:**
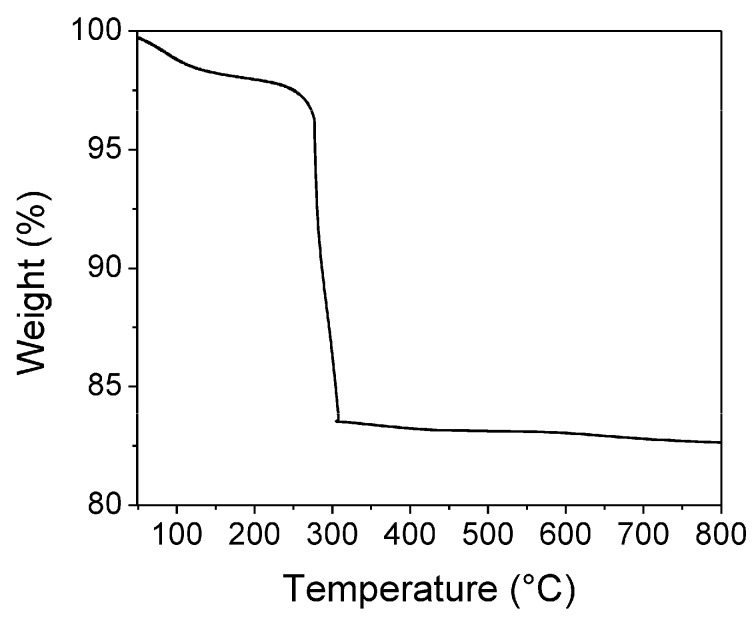
TGA curve of the precursor powder of ZnMn_2_O_4_.

Figure 3 shows a typical TGA curve obtained from the precursor material. The weight loss of 1.5% observed in the range of 51 to 156 °C could be due to the evaporation of physically absorbed water molecules. The following 14.6% weight loss, from 156 to 310 °C, is attributed to the thermal decomposition of dodecylamine molecules existing in the dried precursor. Finally, the small weight loss observed from 310 to 800 °C is probably due to the complete desorption of nitrate ions [49]. Based on this analysis, a temperature of 500 °C was chosen for the calcination treatment of the sample to ensure complete crystallization.

### 3.2. XRD Results

**Figure 4:**
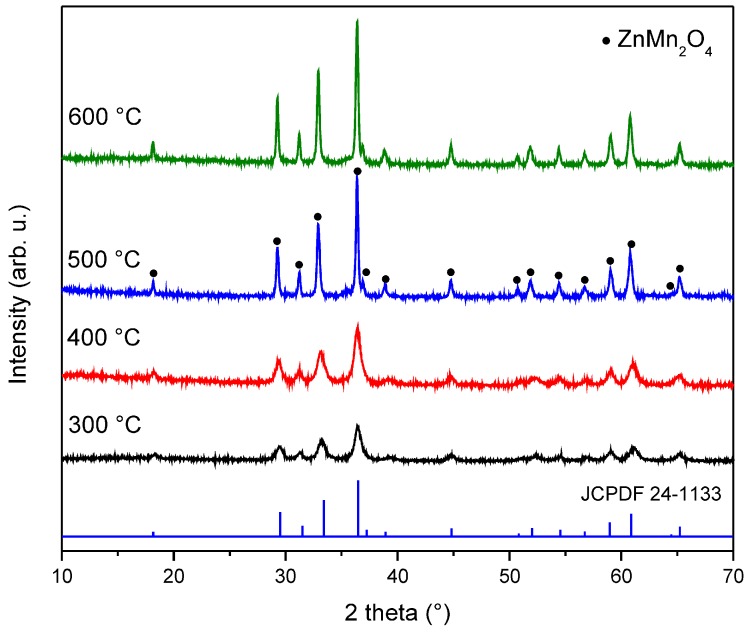
XRD patterns of the ZnMn_2_O_4_ precursor powder after calcination at different temperatures.

Figure 4 shows the crystallinity evolution of the ZnMn_2_O_4_ powder samples calcined at temperatures from 300 to 600 °C. For the samples annealed at 300 and 400 °C, the main peaks corresponding to ZnMn_2_O_4_ were identified using the JCPDF file No. 24-1133; at 500 and 600 °C, a small peak located at 36.9° completely proved the formation of the ZnMn_2_O_4_ spinel structure. An additional feature of the diffractograms at these temperatures was the well-defined narrow peaks that were observed, indicating the high crystallinity of these zinc manganite samples. The crystallite size of the sample calcined at 500 °C was calculated from the most intense peak located at 36.4° using Scherrer´s equation [50], giving a value of 26.6 nm.

### 3.3. Raman Spectroscopy

**Figure 5:**
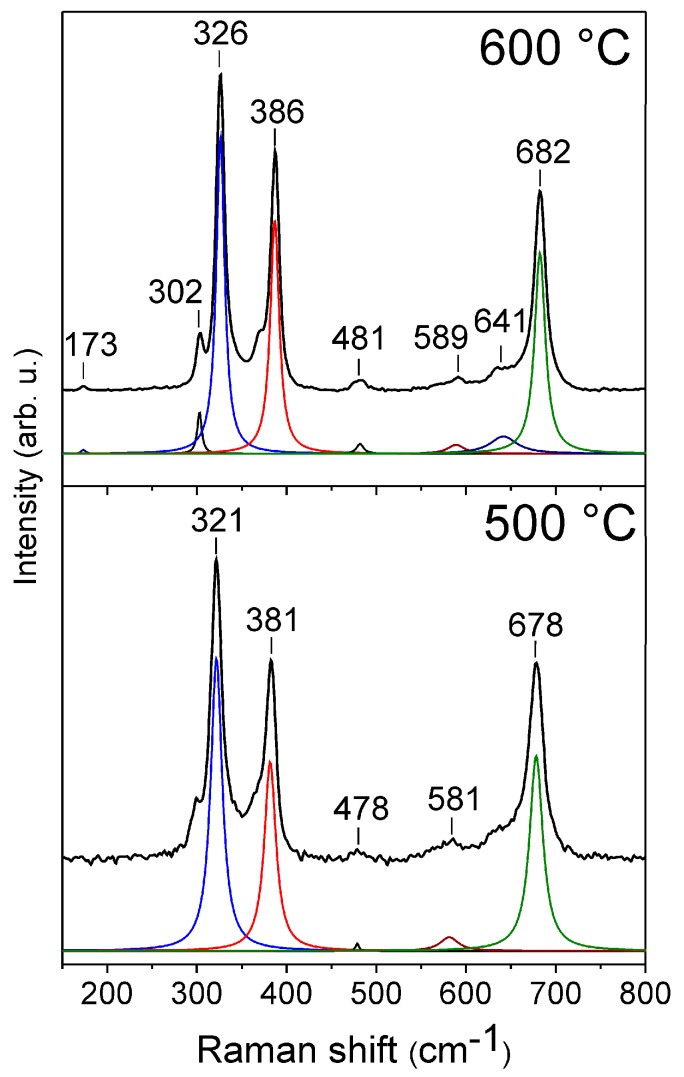
Raman spectra of the ZnMn_2_O_4_ powder calcined at 500 and 600 °C.

Raman spectra of the ZnMn_2_O_4_ powders calcined at 500 and 600 °C are shown in Figure 5. According to group theory, ten Raman active modes (2A_1g_ + 3B_1g_ + 1B_2g_ + 4E_g_) are expected for the tetragonal spinel structure of AMn_2_O_4_ (A = Zn, Mg, Mn), with a space group of I41/amd [51,52]. The Raman spectrum of the powder treated at 500 °C exhibited five Raman vibrational modes, which are the typical modes of the zinc manganite [53,54,55]. It was interesting that eight of the 10 allowed modes were observed for the sample calcined at 600 °C. In this spectrum, a small increase of the intensity and a slight shift of the vibrational modes were observed in contrast with the ones from the spectrum of the sample calcined at 500 °C. The differences between these spectra could be explained by the crystallinity of the ZnMn_2_O_4_ samples, as was confirmed by XRD. According to the literature, it was assumed that modes above 600 cm^−1^ were due to the oxygen motion in the tetrahedral AO_4_ sites, and the low-frequency modes by the octahedral BO_6_ sites [56]; however, this assumption requires further study.

### 3.4. UV-Vis Analysis

**Figure 6:**
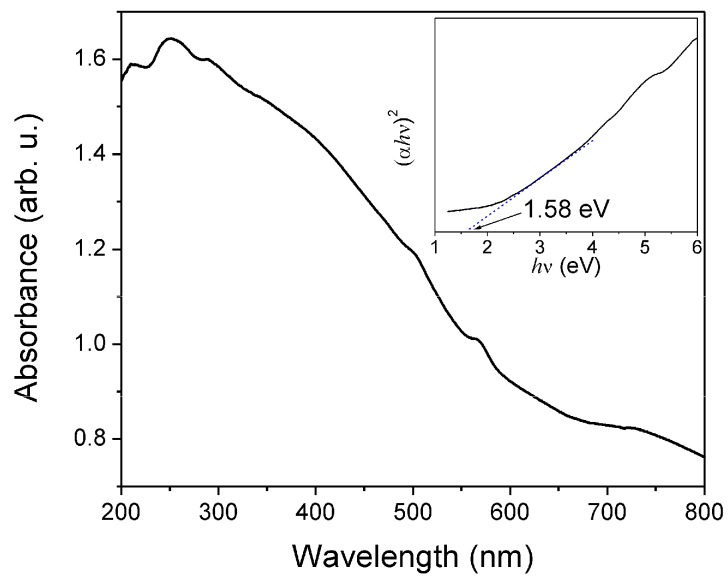
UV-vis spectrum of ZnMn_2_O_4_ nanoparticles.

The optical-absorbance spectrum of the ZnMn_2_O_4_ powder calcined at 500 °C is shown in Figure 6. The ZnMn_2_O_4_ exhibited an absorption band between the 250 and 600 nm. This absorption behavior is characteristic of the ZnMn_2_O_4_ spinel and is in agreement with previous reports [57,58,59]. In order to calculate the band gap energy, Tauc´s formula was used [60,61]. The bang gap energy was determined by extrapolating the linear part of the graph of (αhv)^n^ versus hv up to energy axis at α = 0, where α is the optical absorption coefficient, and n = ½ for indirect transitions and = 2 for direct transitions (see inset of Figure 6). The band gap for the ZnMn_2_O_4_ was of 1.58 eV, considering a direct transition.

### 3.5. TEM Analysis

**Figure 7:**
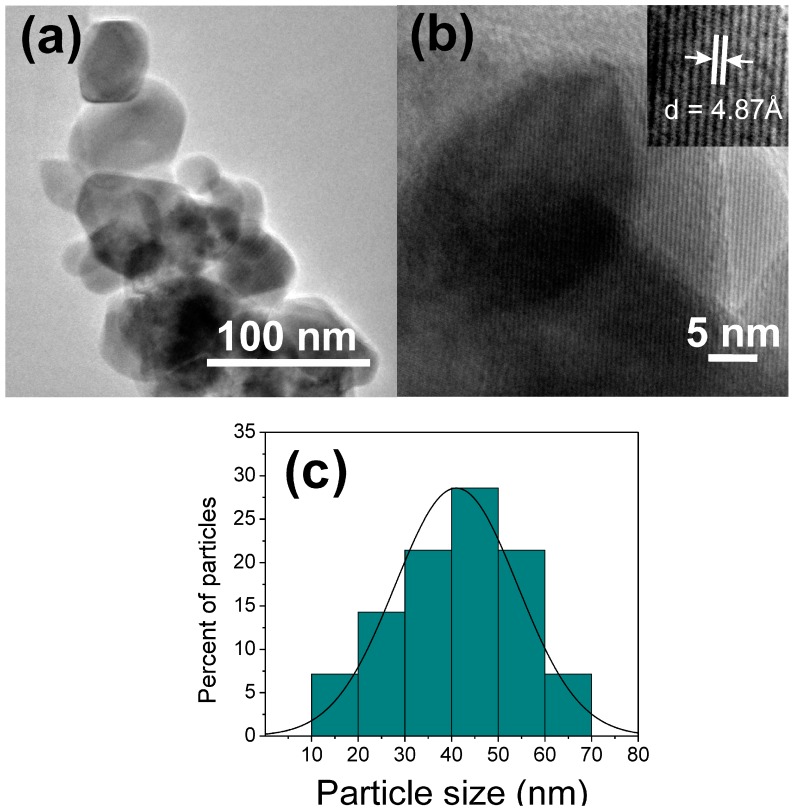
(**a**) TEM and (**b**) HRTEM images of ZnMn_2_O_4_ nanoparticles; (**c**) particle size distribution for the zinc manganite.

Figure 7a,b show TEM and HRTEM images of the zinc manganite powder calcined at 500 °C. The TEM image confirmed the presence of nanoparticles with different size and irregular shapes (Figure 7a). HRTEM further revealed that the nanoparticles were of crystalline nature with an inter-planar d-spacing of 4.87 Å, corresponding to the (101) plane of the ZnMn_2_O_4_ spinel structure (Figure 7b). Figure 7c shows the particle size distribution of the ZnMn_2_O_4_ nanoparticles. The particles were in the range from 10 to 70 nm, with a representative particle size of ~41 nm and a standard deviation of ±13.1 nm. Concerning to the formation mechanism of the nanoparticles, it is known that in colloidal media, nanoparticles are formed by a mechanism of nucleation and growth [62,63]. The formation of ZnMn_2_O_4_ nanoparticles should be produced through these mechanisms, where the nucleation could occur when the manganese nitrate solution was added to the zinc–dodecylamine solution and the particle growth during the agitation of the solution [44]. The dodecylamine also plays an important role in the size and morphology formation of inorganic materials [44,46].

### 3.6. Gas Sensing Performance

**Figure 8:**
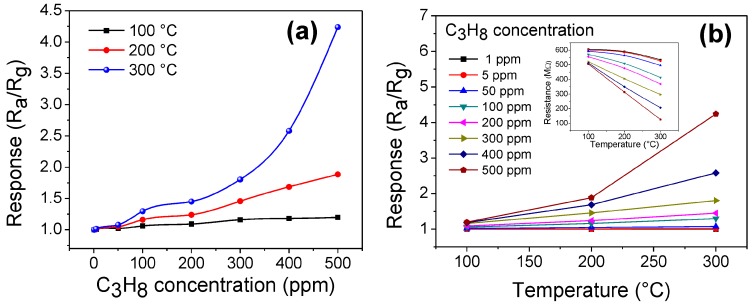
Sensing response of the ZnMn_2_O_4_ nanoparticles as a function of (**a**) C_3_H_8_ concentration and (**b**) operation temperature.

In order to know the capacity of ZnMn_2_O_4_ for its possible application as a gas sensor, the oxide was studied in C_3_H_8_ and CO atmospheres. Figure 8 shows the results obtained in propane atmospheres at different concentrations (1–500 ppm) and operating temperatures (100, 200, and 300 °C).

According to these results, the pellets of ZnMn_2_O_4_ are clearly sensitive to C_3_H_8_ at the given temperature. As expected, the response of the material also increases as the temperature and concentration of propane increase. The good response of the oxide is due to the increase in the number of molecules of the gas that react with the oxygen present on the surface of the pellets. In addition, due to the effect of temperature (when it is increased), there are changes in the electrical resistance [12], causing an increase in the response of the material. Several authors have reported that temperature plays a key role in increasing the efficiency of a material like the one used in this work [9,10,11,12]. In particular, at 100 °C, the oxide showed a poor response: 1.19 at 500 ppm. In contrast, at 200 °C, a slight increase was obtained: 1.88 at the same concentration of propane (see Figure 8a,b). The low response at 100 and 200 °C is due to the fact that the thermal energy is not enough to produce the oxygen desorption reaction [10,17], causing no significant changes in the electrical resistance of the material during the detection test. In contrast, upon increasing the temperature to 300 °C, the response of the pellets increased considerably to 4.23 at 500 ppm of C_3_H_8_ (see Figure 8a). The good response obtained at this temperature is attributed to the fact that during the adsorption of propane, it subsequently reacts with the oxygen present on the surface of the material [9,10], inducing a high interaction between the gas and the surface of the zinc manganite pellets. The mechanism that involves detection in propane atmospheres has not been fully studied. However, it has been suggested in the literature that C_3_H_8_ molecules react with chemisorbed O^−^ species, producing CO_2_, water vapor, and an electron release to the material’s surface [64], causing the changes in the material’s electrical resistance shown in Figure 8a,b. Other authors have studied the chemical interaction between the surface of a material and the molecules of the C_3_H_8_, suggesting some chemical processes that involve the adsorption–desorption of propane, and obtaining results such as those presented in this work [11,65,66].

**Figure 9:**
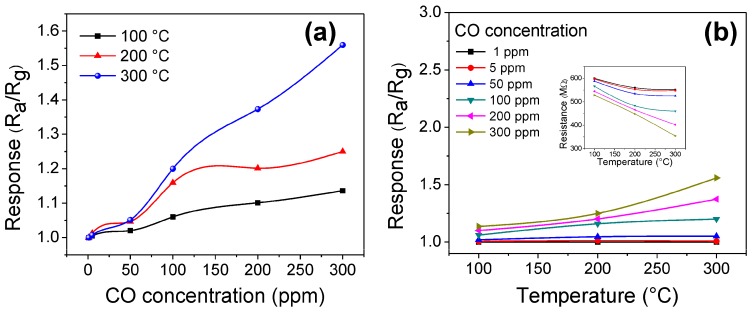
Response of the ZnMn_2_O_4_ sensor as a function of: (**a**) CO concentration and (**b**) working temperature.

Similar trends were obtained when the pellets of ZnMn_2_O_4_ were exposed to atmospheres of CO at different concentrations (1–300 ppm) and working temperatures (100, 200, and 300 °C). The results obtained are shown in Figure 9a,b.

As was the case in propane atmospheres, the zinc manganite pellets showed changes in electrical resistance when the CO molecules contacted the surface of the material. The increase in response to this atmosphere clearly depends on the increase in operating temperature and the concentration of CO. It is known that when a semiconductor metal oxide, like the ZnMn_2_O_4_, is exposed to a gas at a moderate temperature, its electrical resistance is altered due to the reaction of the gas with pre-adsorbed oxygen species [67]. Hence, when performing the tests at 100, 200, and 300 °C, the adsorbed CO reacts with the oxygen present, as a result of temperature, causing an increase in the sensitivity of the material. Therefore, the response obtained in CO was estimated at 1.13 and 1.25 for 300 ppm of CO at temperatures of 100 and 200 °C, respectively (see Figure 9a,b). The maximum response in CO was of 1.55, which corresponds to a concentration of 300 ppm of CO at a temperature of 300 °C (see Figure 9a). Recently, it has been reported in the literature that the increase in the response of a material such as the one used in this work is associated with the increase of oxygen desorption at elevated temperatures [11,12,65]. According to Barsan and Weimar [68], the nature of the adsorbed oxygen species on a semiconductor gas sensor depends on the working temperature: Below 150 °C, the molecular O_2_^−^ species are present; above 150 °C, the ionic O^−^ and O^2−^ species are found. In addition, it is known that the formation of ionic species at high temperatures increases the gas–solid interaction because of the higher reactivity of these species; therefore, the gas response is improved [10,11,17], as happened in this work (see Figure 9).

Considering our results, the zinc manganite was sensitive, as expected, to both changes in C_3_H_8_ and CO concentrations, and operation temperatures. In fact, the high sensitivity of the ZnMn_2_O_4_ sensor was mainly due to the nanometric particle size, since the surface-to-volume ratio of the nano-sized semiconductor is very high; therefore, more active sites increase the sensor response [1,2,18]. In particular, our propane sensitivity results were compared with similar metal oxides, showing that we have succeeded in obtaining a better response. For example, the SnO_2_ and undoped ZnO thin films exhibited a maximum sensitivity of 0.7 and 2.3, respectively; both sensitivities were measured at 300 °C in 300 ppm of C_3_H_8_ [69,70]. The oxide ZnSb_2_O_6_ showed a maximum sensitivity of 1.2 in 300 ppm of C_3_H_8_ at 250 °C [71].

## 4. Conclusions

Zinc manganite nanoparticles were successfully synthesized via a microwave-assisted colloidal route. This simple and economical synthesis process can be extended for the synthesis of nanoparticles of many other simple and complex compounds. Single-phase ZnMn_2_O_4_ was found at 500 °C with an optical band-gap energy of 1.58 eV. Eight Raman vibrational modes were registered from zinc manganite. ZnMn_2_O_4_ nanoparticles with an average particle size of 41 nm were obtained. Test sensors based on these nanoparticles exhibited a good response to C_3_H_8_ and CO at an operating temperature of 300 °C. Based on the results of this work, the ZnMn_2_O_4_ nanoparticles can be considered a promising new material for the sensing of relatively low concentrations of propane.
